# Therapeutic effects of hirsutella sinensis on the disease onset and progression of amyotrophic lateral sclerosis in SOD1^G93A^ transgenic mouse model

**DOI:** 10.1111/cns.13182

**Published:** 2019-07-18

**Authors:** Hai‐Yan Shang, Jing‐Jing Zhang, Zhen‐Fa Fu, Yu‐Fei Liu, Song Li, Sheng Chen, Wei‐Dong Le

**Affiliations:** ^1^ Center for Clinical Research on Neurological Diseases, the First Affiliated Hospital Dalian Medical University Dalian China; ^2^ Liaoning Provincial Key Laboratory for Research on the Pathogenic Mechanisms of Neurological Diseases, the First Affiliated Hospital Dalian Medical University Dalian China; ^3^ General Hospital of Yangtze River Shipping Wuhan Brain Hospital Wuhan China; ^4^ Chifeng Municipal Hospital Chifeng China; ^5^ Department of Neurology, Ruijin Hospital Shanghai Jiao Tong University School of Medicine Shanghai China

**Keywords:** amyotrophic lateral sclerosis, hirsutella sinensis, microglia, motor neuron, neuroinflammation, SOD1^G93A^

## Abstract

**Aims:**

Although the pathophysiology of amyotrophic lateral sclerosis (ALS) is still not completely understood, the deregulated microglia polarization and neuroinflammation have been shown to contribute to the pathogenesis and progression of this disease. In the present study, we aimed to determine whether hirsutella sinensis (HS) could reduce neuroinflammatory and pathological changes in the spinal cord of SOD1^G93A^ model mice of ALS and consequently ameliorate disease onset and progression.

**Methods:**

SOD1^G93A^ mice were chronically treated with HS by gavage. Their lifespan was recorded, and motor behavior was evaluated by rotarod test. The pathological changes in skeletal muscles and motor neurons in spinal cords were assessed by immunofluorescent staining and hematoxylin‐eosin staining. The microglia activation and neuroinflammation were determined by immunofluorescent staining and RT‐PCR.

**Results:**

Our data suggested that repeated HS administration prolonged the lifespan and extended disease duration of ALS mice without significant delay on disease onset. HS ameliorated the pathological changes in the motor neurons and gastrocnemius muscles. Moreover, HS promoted the transition of microglia from pro‐inflammatory M1 to anti‐inflammatory M2 phenotype in the spinal cord of ALS mice.

**Conclusion:**

All these findings indicate that HS may serve as a potential therapeutic candidate for the treatment of ALS.

## 1 INTRODUCTION

Amyotrophic lateral sclerosis (ALS) is a progressive and fatal neurodegenerative disease, characterized by the selective deterioration of upper and lower motor neurons in the motor cortex, brain stem, and spinal cord, leading to muscle paralysis, respiratory deficiency, and death.^1^ The majority of ALS cases (over 90%) are sporadic (sALS), and approximately 10% cases are familial (fALS).^2^ Mutations of the superoxide dismutase‐1 (SOD1) gene account for approximately 20% of fALS. SOD1^G93A^ (glycine 93 to alanine) is one of the common diseases causing gene mutations in ALS, and the SOD1^G93A^ transgenic mouse is the most used experimental model.^2^ Several pathogenic events, including protein misfolding/aggregation, oxidative stress, endoplasmic reticulum (ER) stress, and neuroinflammation, have been found to be involved in ALS pathogenesis. However, the exact pathophysiology of ALS is still not fully understood. Consequently, till now, only two drugs, riluzole and edaravone, have been approved by FDA and applied clinically for the treatment of ALS. These two drugs moderately prolong the lifespan of patients with ALS. However, riluzole benefits only a subset of patients, and its side effects are difficult to predict. Edaravone, as a free radical scavenger, appears to show modest benefits on slowing disease progression in patients with short disease duration and good respiratory function.^3^, ^4^ Therefore, new therapeutic approaches are still urgently needed.

Recently, the microglia‐mediated inflammation has been recognized as a neuropathological hallmark of ALS.^5^ Microglia cells, the resident tissue macrophages of the central nervous system (CNS), belong to the innate immune system. During the development, adulthood and aging of CNS, microglia play many critical roles in not only the mediation of immune response, but also the elimination of apoptotic cells, production of growth factors, maintenance of synapse structure and function. However, the excessive production of pro‐inflammatory cytokines from over‐activated microglia contributes to the pathophysiology progression in many neurodegenerative diseases, including ALS.^6^, ^7^ Therefore, suppression of microglia‐mediated inflammation has been considered as an important strategy in ALS treatment.

**Figure 1:**
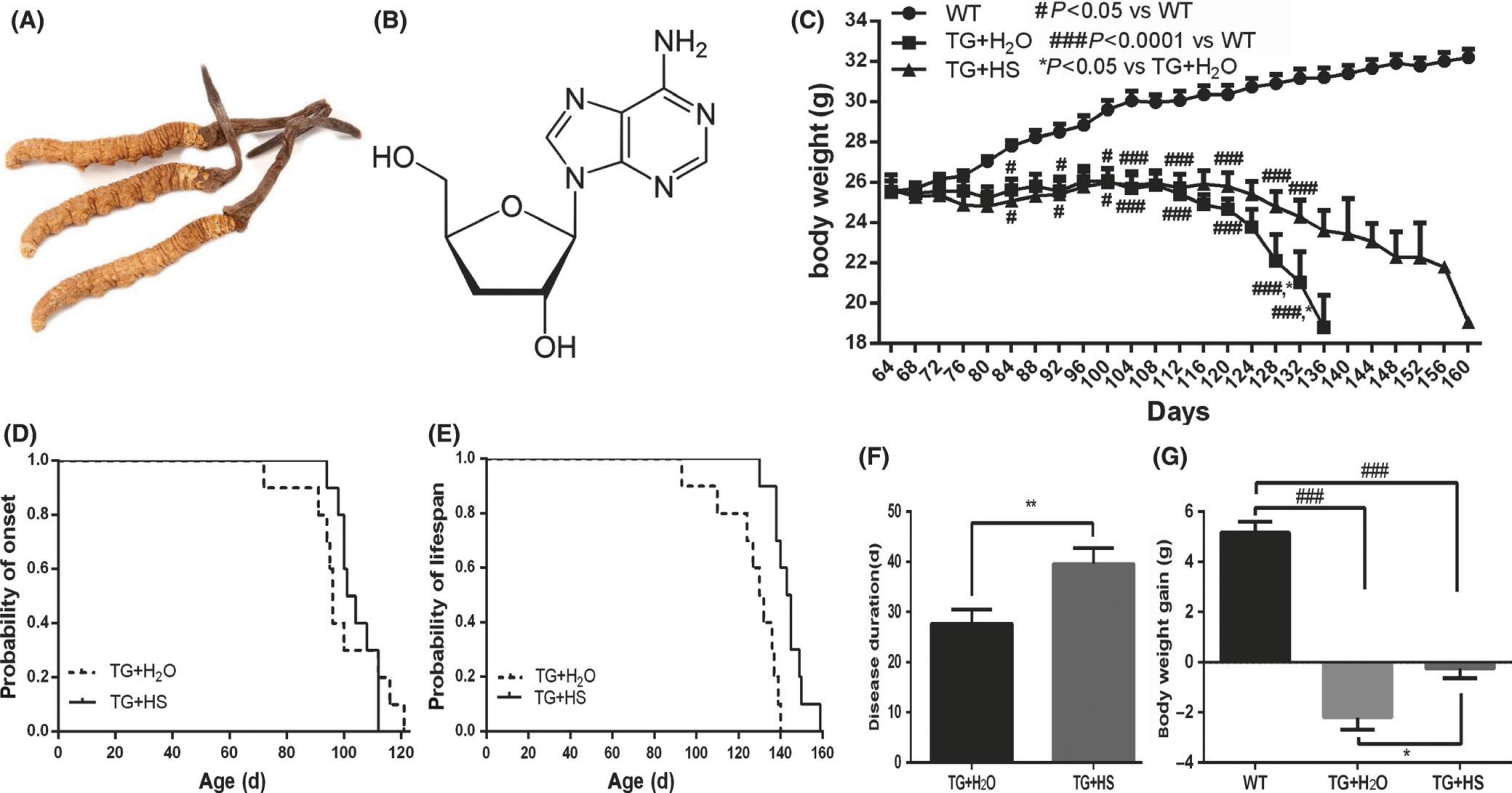
The effects of hirsutella sinensis (HS) on body weight, disease onset, lifespan, and duration in SOD1^G93A^ mice. A, Raw material of HS; B, chemical structure of main component of HS, cordycepin; C, body weight curves of three groups. The probability of disease onset (D) and lifespan (E) was analyzed by Kaplan‐Meier survival analysis; F, the data of disease duration. G, Body weight gain during disease progression (between 64 and 124 days). *P* values were analyzed by one‐way ANOVA. Data are presented as mean ± SEM. **P < 0.01 vs TG + H_2_O group; ^###^ *P* < 0.0001 vs wild‐type (WT) group;*P < 0.05 vs TG + H_2_O group. N = 10 in each group

It has been reported that traditional Chinese medicine might be beneficial to improve symptoms and prolong the survival of patients with ALS.^8^, ^9^, ^10^ Hirsutella sinensis (HS, also known as Cordyceps sinensis, Chinese caterpillar fungus or “Dong Chong Xia Cao” in Chinese, Figure 1A) is a unique fungus growing on caterpillars and is a highly valued tonic medicine claimed to treat a wide range of disorders, such as asthma,^11^ chronic kidney disease,^12^ and kidney transplant recipients.^13^ Cordycepin (3′‐deoxyadenosine), one of the major chemical components of HS (Figure 1B), has been identified to exert anti‐oxidative, anti‐inflammation, antitumor, and neuroprotective effects.^14^, ^15^, ^16^, ^17^ Moreover, previous studies have further revealed a suppressive role of cordycepin on LPS‐induced microglia activation and inflammation in vitro.^18^, ^19^ Adenosine, another active component of HS, is not only an energy transfer and signal conversion agent in cells, but also plays a role in cell protection and tissue damage prevention. It has been shown that adenosine could regulate neuroimmunity and treat mental disorders.^20^ HS is rich in a variety of chemical components that have medicinal activities,^21^ suggesting a therapeutic potential of HS for ALS. To confirm this hypothesis, in this study, we evaluated the impacts of repeated HS administration on disease onset and progression of ALS in SOD1^G93A^ mouse model. The potential ameliorating activities of HS on various disease phenotypes, especially the microglia activation and neuroinflammation, were further investigated.

## 2 MATERIALS AND METHODS

### 2.1 Animals and treatments

SOD1^G93A^ transgenic (TG) mice, carrying mutant human SOD1 with a substitution of glycine to alanine in position 93, are the most commonly used ALS animal model. Male SOD1^G93A^ mice (B6SJL‐Tg‐SOD1*G93A‐1Gur/J, stock number 002726) were purchased from Jackson Laboratory and then bred to wild‐type (WT) females from the same background to get the offspring. The genotypes of the offspring were determined by PCR of DNA extracted from tail tissues. All mice were kept in constant temperature and controlled lighting/dark cycle (12/12 hours, light on 7 am). All animals care and experimental procedures were carried out in accordance with the Laboratory Animal Care Guidelines approved by the Institutional Animal Care Committee at Dalian Medical University.

Male SOD1^G93A^ TG mice were randomized into two groups: (a) TG + H_2_O group (n = 28), treated with drinking water by gavage (10 mL/kg body weight, once daily); and (b) TG + HS group (n = 28), treated with HS by gavage (1 g/kg body weight, 10 mL/kg, once daily). The equivalent dosage of HS for mice was converted from clinical dosage for human patients according to previous method.^22^ HS (Corbrin capsule, Cs‐C‐Q80) was obtained from Hangzhou Sino‐American Pharmaceutical Co. Ltd. The content of two Corbrin capsules (200 mg/cap) was dissolved in 4 mL drinking water.^23^ Twenty‐eight age‐matched WT littermates were administered with drinking water by gavage (once daily, 10 mL/kg body weight). All mice were weighed every 4 days from 64 days of the age to death. Eighteen mice per group were sacrificed at the age of 120 days for fresh spinal cords and muscles sampling or were perfused with PFA for the histological analysis of spinal cords.

### 2.2 Rotarod test for the assessment of disease onset

From the age of 70 days, all animals were subjected to rotarod training using an IITC rotarod apparatus (4 cm diameter, 20 rpm; IITC Life Science Inc). After 7 days adaptation training (once daily, 5 minutes/d), the mice were tested every 2 days with three trials (5 minutes/trial). The rotarod performance was recorded to assess whether animals could complete one of all three trials. While the mice could not stay on the rotating rod for 5 minutes for all three trials, the date of the age was recorded and defined as disease onset.^24^

### 2.3 Assessment of animal's lifespan

The date was considered as the day of death, when the mice could not right itself within 30 seconds after being placed on its back.^25^ Then, the lifespan was calculated as the duration between the day of birth and the day of death, and the disease duration was defined as the duration between the day of disease onset and the day of death.

### 2.4 Immunofluorescent staining

At the age of 120 days, mice were anesthetized and perfused with ice‐cold 100 mmol/L phosphate‐buffered saline (PBS, pH 7.4) and then 4% paraformaldehyde (PFA). The spinal cords were removed, post‐fixed in 4% PFA overnight at 4°C, and then transferred to 15% and 30% sucrose in PBS for 24 hours. The tissues were coated with optimal cutting temperature compound (OCT, Tissue‐Tek, 4583; SAKURA). The fixed L4‐5 spinal cords were cut with a Leica cryostat (CM‐1950S; Leica) to 10 μm thickness, mounted on gelatin‐coated slices, and stored at −80°C when needed.

The slices of spinal cords were roasted at 55°C and washed with PBS for three times. Then, the slides were incubated in 5% bovine serum albumin in PBS containing 0.3% Triton X‐10 and 0.05% sodium azide for 1 hour and incubated at 4°C overnight with the following primary antibodies: SMI‐32 antibody (sc‐133165, 1:50; Santa Cruz), ionized calcium binding adapter molecule 1 (Iba‐1) antibody (019‐19741, 1:1000; Wako), glial fibrillary acidic protein (GFAP) antibody (Z0334, 1:1000; Dako), cluster of differentiation 86 (CD86) antibody (553689, 1:1000; BD Biosciences), and arginase‐1 (Arg‐1) antibody (sc‐271430, 1:40; Santa Cruz). After the primary antibodies being removed, the slices were washed with PBS and incubated with a proper secondary antibody: anti‐rabbit IgG (H + L), F(ab′)2 Fragment (Alexa Fluor 594/488 Conjugate; 8889S/4412S, 1:2000; Cell Signaling), anti‐mouse IgG (H + L), F(ab′)2 Fragment (Alexa Fluor 594/488 Conjugate; 8890S/4408S, 1:2000; Cell Signaling), or Cy3 Goat Anti‐Rat IgG (H + L; A0507, 1:2000; Beyotime), for 2 hours. Pictures were visualized and photographed by a fluorescent microscope equipped with DP80 CCD digital camera (Olympus). The integrated density of positive staining was measured by Image J software (https://imagej.nih.gov/ij/) on 10 slices per mouse (n = 6 in each group).

### 2.5 Motor neuron survival analysis

The embedded lumbar L4‐5 spinal cords were cut (10 μm thickness) with a Leica cryostat (CM‐1950S; Leica), mounted on gelatin‐coated slices, and stored at −80°C until needed. Total 50 slices per mouse (one slice from every four consecutive slices) were selected for Nissl staining. Briefly, sections were immunofluorescently stained with 1% cresyl violet (Sigma‐C5042; Sigma‐Aldrich) for 10 minutes and then were dehydrated in gradient alcohol and cleared in xylol. The stained slices were observed and photographed using microscope (Olympus). The motor neurons in the anterior horns of both sides were examined by a technician who was blinded to the experimental design. The motor neurons were counted with following criteria: (a) the neurons in the anterior horn and in the ventral tip of the central canal; (b) the neurons with a maximum diameter ≥20 μm; and (c) the neurons with a distinct nucleolus.^26^

For SMI‐32 immunofluorescence staining, twenty slices (10 μm thickness) of the L4‐5 spinal cord were selected at an interval of 10 slices per mouse. The slices were stained with SMI‐32 antibody according to the method of immunofluorescence staining and captured by the microscope (Olympus). Motor neurons in anterior horn of each slice were observed and counted in a blinded manner.

### 2.6 Pathological analysis of skeletal muscles

Fresh gastrocnemius muscle (5 × 5 × 10 mm^3^) of mice at 120 days of age was dissected, embedded with bassora gum, and immersed immediately in liquid nitrogen. The muscle tissues were sectioned at 10 μm thickness and stained with hematoxylin‐eosin (HE) for morphological analysis or with nicotinamide adenine dinucleotide hydrogen (NADH) to determine muscle fiber types.

### 2.7 Real‐time PCR

Mice at the age of 120 days were sacrificed for spinal cord tissue sampling. The total RNA was extracted using RNAiso Plus (Total RNA extraction reagent; Takara). Then, the total RNA was synthesized for cDNA by Revertra Ace qPCR RT kit (Takara). Real‐time PCR was performed with TransStart Top Green qPCR SuperMix (TransGen Biotech) and measured by Applied Biosystems 7500 Real‐Time PCR System (Life Technologies Corporation). The primer sequences used were summarized in Table 1. The relative expression levels were analyzed by the 2^−∆∆C^ *^t^* algorithm normalizing to GAPDH and relative to the control groups (n = 6 in each group).

**Table 1:** Primer sequences for RT‐PCR

Name	Forward (5′‐3′)	Reverse (5′‐3′)
GAPDH	TGTGTCCGTCGTGGATCTGA	TTGCTGTTGAAGTCGCAGGAG
IL‐1β	AAGGGGACATTAGGCAGCAC	ATGAAAGACCTCAGTGCGGG
TNF‐α	CCAGTGTGGGAAGCTGTCTT	AAGCAAAAGAGGAGGCAACA
CD86	ACGATGGACCCCAGATGCACCA	GCGTCTCCACGGAAACAGCA
Arg‐1	CTTGCGAGACGTAGACCCTG	TCCATCACCTTGCCAATCCC
CD206	TCAGCTATTGGACGCGAGGCA	TCCGGGTTGCAAGTTGCCGT

### 2.8 Statistical analysis

Data of disease onset and lifespan were analyzed by Kaplan‐Meier survival analysis in Statistical Product and Service Solutions (SPSS; version 20.0). Data distribution was examined by a Shapiro‐Wilk test. If the data meet normal distribution and the variances between groups were equal, one‐way analysis of variance (ANOVA) with or without Bonferroni post hoc multiple comparisons was performed using GraphPad Prism (version 7; GraphPad Software Inc); otherwise, nonparametric tests (Mann‐Whitney *U* test or Kruskal‐Wallis analyses) were used. All data were presented as mean ± SEM. The statistical analysis results were considered significant when *P*‐value was <0.05.

## 3 RESULTS

### 3.1 Impacts of HS on body weight, disease onset, lifespan, and disease duration in SOD1^G93A^ mice

Body weight loss is a frequent feature of ALS. As shown in Figure 1C,G, while the body weight of WT mice was gradually increased, SOD1^G93A^ mice showed an clearly decreased body weight during disease progression (between 64 and 124 days). Repeated HS treatment ameliorated this body weight loss in SOD1^G93A^ mice (Figure 1G).

The rotarod test was conducted to measure the motor function of animals and to determine the date of disease onset. As shown in Figure 1D, compared with drinking water‐treated SOD1^G93A^ mice, HS‐treated SOD1^G93A^ mice showed no significant delay of disease onset (104.1 ± 2.1 vs 99.3 ± 4.5, *P* > 0.05). However, HS treatment significantly extended the lifespan of SOD1^G93A^ mice (143.7 ± 2.5 vs 126.8 ± 4.7, **P < 0.001, Figure 1E). Additionally, the disease duration of HS‐treated mice was significantly prolonged (39.6 ± 3.1 vs 27.7 ± 2.8, **P < 0.001, Figure 1F).

### 3.2 HS ameliorated motor neuron loss in spinal cord of SOD1^G93A^ mice

**Figure 2:**
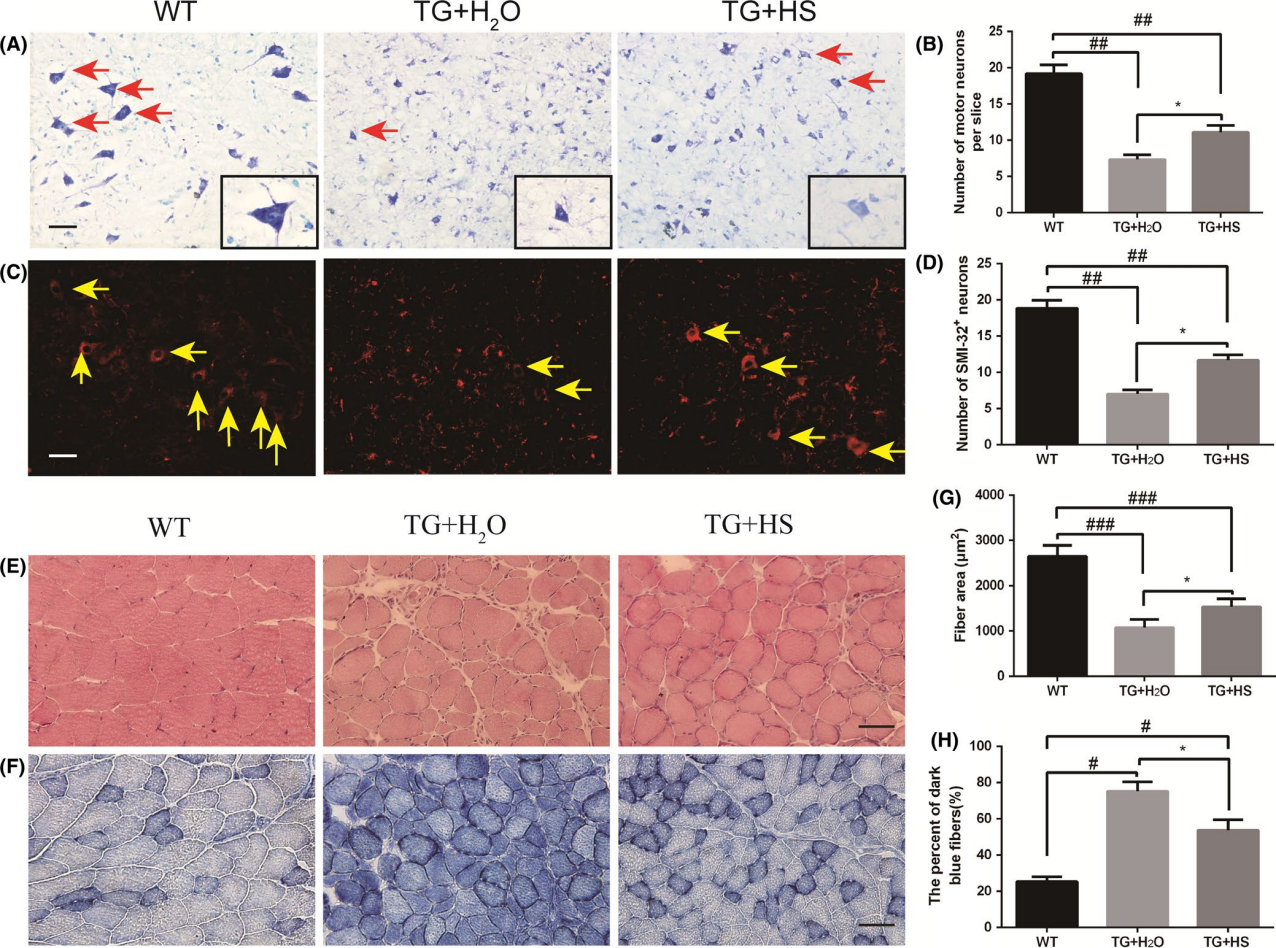
Impacts of hirsutella sinensis (HS) on motor neuron survival and pathological changes in gastrocnemius muscles in SOD1^G93A^ mice. A, The photomicrographs of motor neurons in the anterior horn of spinal cords of mice evaluated by Nissl staining; scale bar = 50 μm; B, the counts of motor neurons in the anterior horn of L4‐5 segments by Nissl staining (average of each slice of total 50 sections per mouse, N = 6); C, the photomicrographs of motor neurons in the anterior horn of spinal cords of mice by SMI‐32 immunofluorescent staining; D, the number of SMI‐32 positive motor neurons in both sides of one slice in the spinal cords. ^##^ *P* < 0.01 as compared with wild‐type (WT) group; *P < 0.05 vs TG + H_2_O group. N = 6 in each group. The pathological alterations of gastrocnemius muscles in SOD1^G93A^ mice were determined by HE staining (E) and NADH staining (F); scale bar = 50 μm. G, The results of muscle fiber area in gastrocnemius muscles. H, The percent of the number of dark blue fibers (%). *P* values were analyzed by one‐way ANOVA. ^###^ *P* < 0.0001 as compared with WT group; ^#^ *P* < 0.05 vs TG + H_2_O group; *P < 0.05 vs TG + H_2_O group. N = 6 in each group

Nissl staining was performed to investigate the effects of HS on motor neuron loss in the L4‐5 spinal cord of SOD1^G93A^ mice. As shown in Figure 2A,B, loss of motor neurons was observed in SOD1^G93A^ mice as compared with the WT mice (7.70 ± 0.49 vs 18.0 ± 1.02, ^##^ *P* < 0.001). This declined motor neuron survival can be partially ameliorated by HS treatment (10.9 ± 0.61 vs 7.70 ± 0.49, *P < 0.05).

Moreover, immunofluorescent staining with neuron‐specific biomarker SMI‐32 further confirmed the neuroprotective effect of HS. As shown in Figure 2C, motor neurons in the anterior horn of SOD1^G93A^ mice were smaller and fewer than those of WT mice. These morphological changes and motor neuron loss in the anterior horn of spinal cord of SOD1^G93A^ mice can be partially reversed by HS treatment. Statistical analysis further indicated that the total counts of SMI‐32‐positive motor neurons per section in HS‐treated SOD1^G93A^ animals were clearly increased as compared with the control mice (11.4 ± 0.87 vs 6.89 ± 0.69, *P < 0.05, Figure 2D).

### 3.3 HS relieved pathological changes in gastrocnemius muscles of SOD1^G93A^ mice

As shown in the Figure 2, at the age of 120 days, the structures of muscle fibers changed apparently in SOD1^G93A^ mice as compared with those in WT littermates. The HE‐stained cryosections of gastrocnemius muscles in SOD1^G93A^ mice illustrated typical pathological features of ALS including atrophic muscle fibers, hematoxylin inclusion, and central nuclei (Figure 2E). Our data clearly showed that HS treatment ameliorated the muscle atrophy by increasing fiber area (Figure 2G).

Furthermore, we also performed NADH staining to quantify the grouped type I or type II myofibers and to evaluate the oxidative metabolism in gastrocnemius muscles. Consistent with HE staining, the NADH staining showed more dark blue oxidative muscle fibers and grouped myofibers in SOD1^G93A^ mice than WT mice (Figure 2F). We found that HS treatment ameliorated these pathological changes (Figure 2F,H).

### 3.4 Effects of HS on microglial and astrocytic activation in the spinal cord of SOD1^G93A^ mice

**Figure 3:**
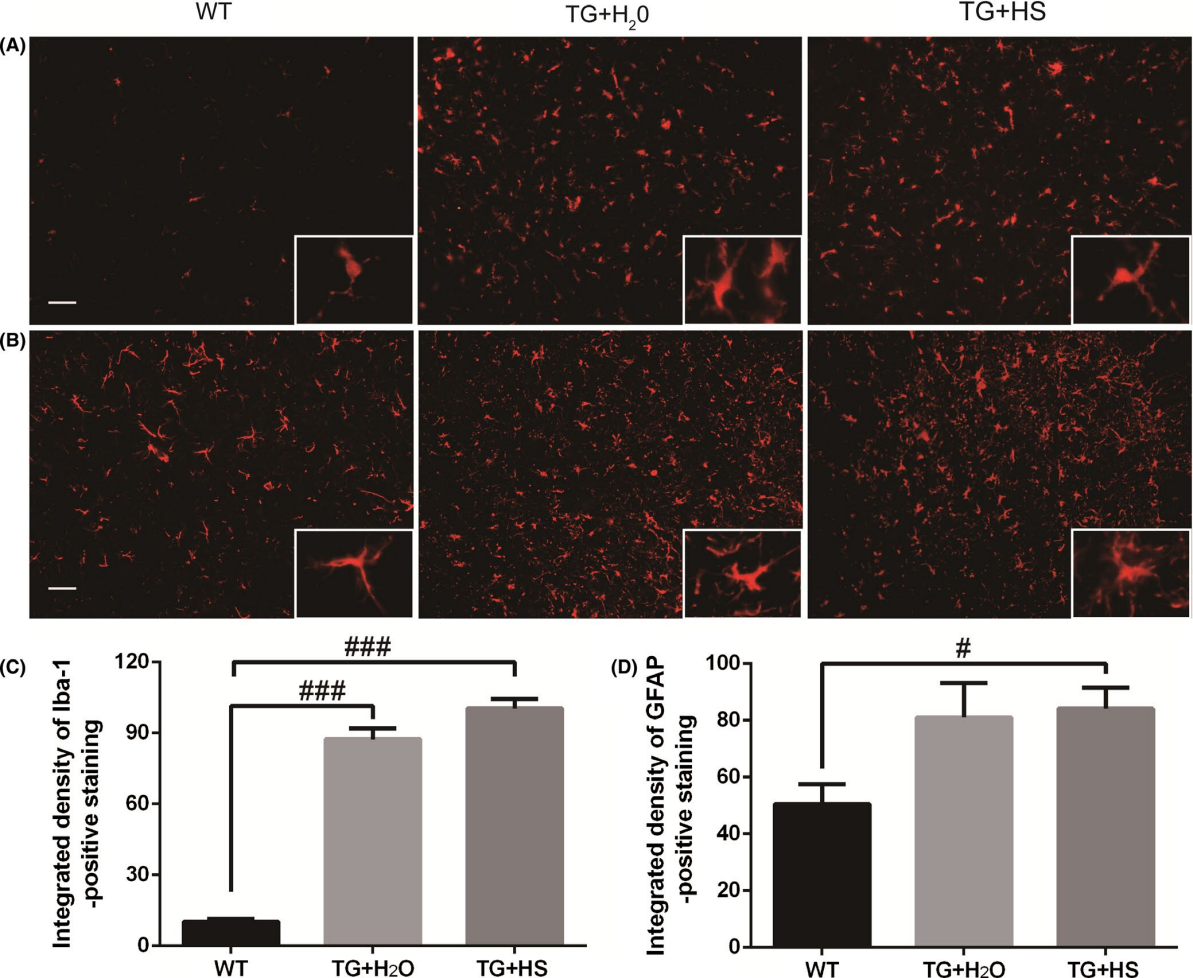
Effects of Hirsutella sinensis on the activation of microglia and astrocytes. Iba‐1 and GFAP antibodies were used to detect the microglia (A) and astrocytes (B), respectively, in the L4‐5 spinal cords, scale bar = 50 μm. The integrated density of Iba‐1‐positive staining (C) and GFAP‐positive staining (D) was quantitatively analyzed by one‐way ANOVA. N = 6 in each group. Values are presented as mean ± SEM. ^###^ *P* < 0.0001 vs wild‐type (WT) group; ^#^ *P* < 0.05 vs WT group

In order to investigate the effects of HS on microglial and astrocytic activation in spinal cords of mice, the immunofluorescence staining was performed using anti‐Iba‐1 antibody and anti‐GFAP antibody, respectively. As shown in Figure 3A, there were more Iba‐1‐positive cells in spinal cord of SOD1^G93A^ mice than WT mice. Quantitative analysis showed that the integrated density of Iba‐1 in the spinal cord of SOD1^G93A^ mice was obviously increased as compared with WT group, whereas the integrated density in HS‐treated SOD1^G93A^ mice had no significant difference as compared with those control SOD1^G93A^ mice treated with water (Figure 3C). GFAP‐positive staining and quantitative analysis indicated a similar tendency as Iba‐1, although no statistical significance was reached (Figure 3B,D). These imaging data were further confirmed by Western blotting (supplementary Figure S1).

### 3.5 HS rebalanced microglia M1/M2 polarization in spinal cord of SOD1^G93A^ mice

**Figure 4:**
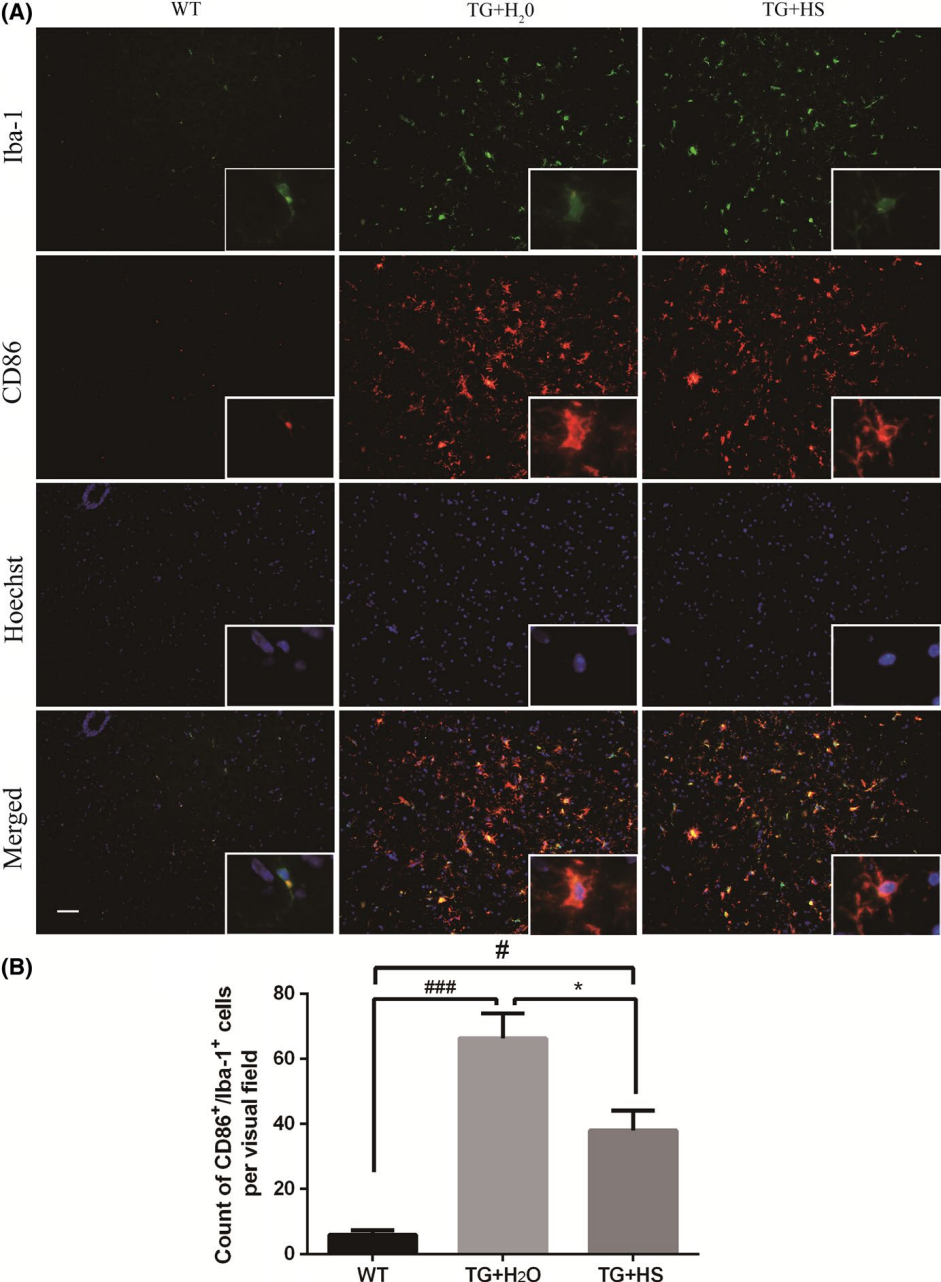
Hirsutella sinensis (HS) decreased the number of CD86^+^/Iba‐1^+^ immunofluorescent staining cells. A, The CD86 (green) and Iba‐1 (red) co‐stained microglia cells in the L4‐5 spinal cords in SOD1^G93A^ mice. B, The cell counts of CD86^+^/Iba‐1^+^ staining by one‐way ANOVA. Scale bar = 50 μm. Data are presented as mean ± SEM. ^###^ *P* < 0.0001 vs wild‐type (WT) group; ^#^ *P* < 0.05 vs WT group; *P < 0.05 vs TG + H_2_O group. N = 6 in each group

**Figure 5:**
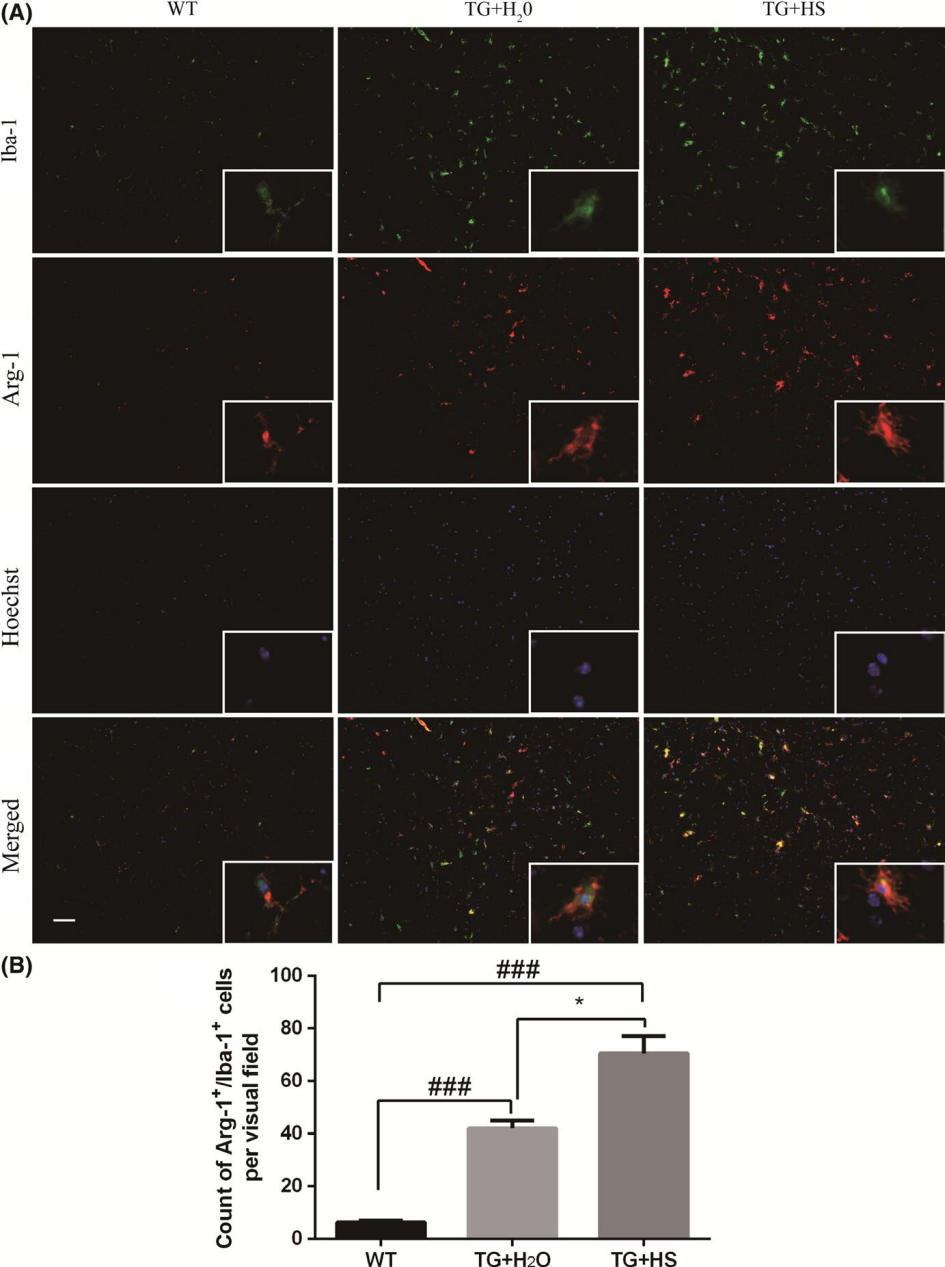
Hirsutella sinensis (HS) elevated the number of Arg‐1^+^/Iba‐1^+^ immunofluorescent staining cells. A, The Arg‐1(green) and Iba‐1(red) co‐stained microglia cells in the L4‐5 spinal cords in SOD1^G93A^ mice. B, The cell count of Arg‐1^+^/Iba‐1^+^ staining. Scale bar = 50 μm. *P* values were analyzed by one‐way ANOVA. Data are presented as mean ± SEM. ^###^ *P* < 0.0001 vs wild‐type (WT) group; *P < 0.05 vs TG + H_2_O group. N = 6 in each group

To investigate whether HS affected the phenotype of microglial activation, double staining was performed in the L4‐5 spinal cord of SOD1^G93A^ mice using CD86 (M1 marker) or Arg‐1 (M2 marker) with Iba‐1. As expected, the counts of CD86^+^/Iba‐1^+^ cells increased in the spinal cord of SOD1^G93A^ mice as compared with WT mice, indicating a stimulated M1 polarization (Figure 4A). Moreover, the total counts of Arg‐1^+^/Iba‐1^+^ cells were also increased in SOD1^G93A^ mice as compared with WT mice (Figure 5A), suggesting a compensatory change in M2 polarization in response to toxic stimuli. Interestingly, while HS treatment significantly inhibited the M1 polarization (Figure 4B), HS further strengthened the M2 polarization of microglia (Figure 5B). These results indicated that HS treatment might promote the transition of microglial polarization from M1 to M2 phenotype.

**Figure 6:**
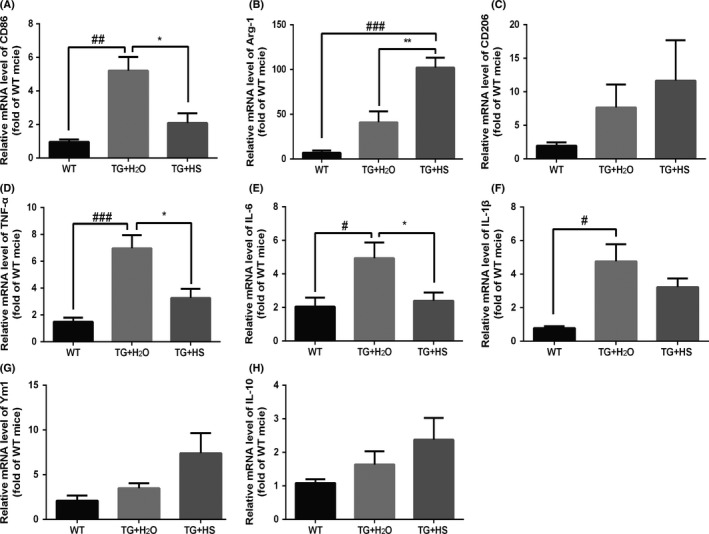
Effects of hirsutella sinensis (HS) on microglia activation and cytokines levels. The relative expression of mRNA of CD86 (A), Arg‐1 (B), and CD206 (C) was analyzed by real‐time PCR by one‐way ANOVA. N = 6 in each group. ^###^ *P* < 0.0001 vs wild‐type (WT) group; ^##^ *P* < 0.001 vs WT group; ^**^ *P* < 0.01 vs TG + H_2_O group; ^*^ *P* < 0.05 vs TG + H_2_O group. The relative expression of TNF‐α (D), IL‐6 (E), IL‐1β (F), Ym1 (G), and IL‐10 (H) in spinal cords was analyzed by real‐time PCR by one‐way ANOVA. N = 6 in each group. Data are presented as mean ± SEM. ^###^ *P* < 0.0001 vs wild‐type (WT) group; *P < 0.05 vs TG + H_2_O group; ^#^ *P* < 0.05 vs WT group

We further performed RT‐PCR to evaluate the mRNA level of M1 and M2 markers in the L4‐5 spinal cord of SOD1^G93A^ mice. Consistent with the data of immunofluorescent staining, the mRNA level of M1 marker CD86 was significantly increased in SOD1^G93A^ mice as compared with WT mice and was clearly reduced by HS treatment (Figure 6A). The mRNA level of M2 marker Arg‐1 was increased in SOD1^G93A^ mice, which was further promoted by HS administration (Figure 6B). The mRNA level of CD206, another M2 marker, shared the similar trend as Arg‐1 (Figure 6C).

### 3.6 HS partially reversed the elevation of mRNA levels of cytokines in the spinal cord of SOD1^G93A^ mice

The mRNA levels of both pro‐inflammatory cytokines (TNF‐α, IL‐6, and IL‐1β) and anti‐inflammatory cytokines (Ym1 and IL‐10) were evaluated in the spinal cord of SOD1^G93A^ mice by RT‐PCR. Our results indicated that, as compared with WT group, the mRNA levels of pro‐inflammatory cytokines were significantly increased in TG mice treated with water and were partially reversed by HS treatment (Figure 6D‐F). Additionally, although no statistical significance was found, the mRNA levels of Ym1 and IL‐10 were also upregulated in those HS‐treated SOD1^G93A^ mice as compared with control TG mice (Figure 6G,H).

## 4 DISCUSSION

It has now become evident that neuroinflammation is a prominent pathological hallmark of neurodegenerative diseases, such as Alzheimer's disease, Parkinson's disease, and ALS. Over‐activated microglia and astrocytes as well as infiltrating T lymphocytes have been identified in ALS experimental models and patients. Therefore, anti‐inflammatory drugs may provide a new strategy for ALS therapy. HS, a natural sourced fungus in traditional Chinese medicine, has been reported to inhibit both canonical and noncanonical inflammasomes^27^ and may have the therapeutic effects against ALS.

Our present study found that HS treatment slowed the disease progression, prolonged the lifespan, and extended the disease duration of SOD1^G93A^ mouse model of ALS. In addition, we demonstrated here that HS treatment could significantly ameliorate the pathological changes in the spinal cord and gastrocnemius muscle. Moreover, our data suggested that the neuroprotective property of HS may be due to antioxidant activity and regulation of microglial activation and neuroinflammation.

Amyotrophic lateral sclerosis is pathologically characterized by the motor neuron loss in the anterior horn of L4‐5 spinal cords. Thus, it is important to evaluate whether HS treatment can reduce the motor neuron loss. Our results indicated that repeat HS treatment apparently ameliorates the motor neuron loss in SOD1^G93A^ mice, suggesting a promising neuroprotective activity. Increasing lines of evidence have shown that neuroinflammation can aggravate the damage of ALS motor neurons.^28^, ^29^ The inflammatory response is accompanied by the activation of microglia and astrocytes, which can further release the pro‐inflammatory factors, binding to the corresponding receptors on the surface of the motor neurons and aggravate the injury and death of motor neurons through a series of signal transduction pathways.^30^ Our data showed that the integrated density of microglia and astrocytes in SOD1^G93A^ model mice were increased as compared with WT control mice, suggesting an inflammatory response in SOD1^G93A^ mice.

The activated microglia can be polarized into M1 and M2 phenotypes. M1 phenotype is the classical activation distinguished by the upregulated expression of not only CD86 but also pro‐inflammatory cytokines causing cell death and tissue damage.^31^ M2 phenotype is normally characterized by the massive expression of scavenger receptor CD206 and arginase 1 (Arg‐1), together with the elevated release of anti‐inflammatory cytokines.^32^ In the early stage of ALS, microglia cells are mainly polarized into M2 phenotype, releasing a large number of anti‐inflammatory factors and exerting neuroprotective effect. However, in the middle and later stages of disease, along with the development and movement course of neuronal damage cumulative, more microglial cells were polarized into pro‐inflammatory and cytotoxic M1 phenotype.^33^, ^34^ Our results showed that, at the age of 120 days, the HS‐treated SOD1^G93A^ mice presented less CD86^+^/Iba‐1^+^ cells but more Arg‐1^+^/Iba‐1^+^ cells in the L4‐5 spinal cord than those H_2_O‐treated SOD1^G93A^ mice. Furthermore, after HS treatment, the relative mRNA expression level of M1 marker CD86 was clearly reduced, whereas the M2 marker Arg‐1 was significantly increased, and CD206 also had the upregulating tendency. These results indicated that HS might promote the transition of microglia polarization from M1 to M2 phenotype.

Previous studies have shown that M1 phenotype microglia are distinguished by increased expressions of pro‐inflammatory cytokines TNF‐α, IL‐6, IL‐1β, and inducible nitric oxide synthase (iNOS), while M2 activation is accompanied by the production of a large number of anti‐inflammatory cytokines such as Ym1 and IL‐10.^31^, ^32^ Previous studies found that HS regulated inflammatory by inhibiting the expression of TNF‐α, IL‐6, and IL‐1β,^27^ and its main component cordycepin suppressed the release of TNF‐α and IL‐1β and exerted neuroprotective effects.^19^ Consistent with these previous findings, we found that the mRNA levels of TNF‐α and IL‐6 were clearly decreased in HS‐treated SOD1^G93A^ mice compared with SOD1^G93A^ mice treated with water. Interestingly, the mRNA levels of Ym1 and IL‐10 had an upregulated tendency after HS administration. These data further validated that HS might inhibit the activation of M1 microglia and promoted the M2 microglia polarization.

Skeletal muscle atrophy is another characteristic of ALS, as the consequence of the motor neuron loss. Our dater showed that HS treatment ameliorated the atrophy of gastrocnemius muscles in SOD1^G93A^ mice. NADH staining is usually used to distinguish muscle fiber types. Type I fibers normally exerted dark blue NADH staining, whereas the type II fibers have the light NADH staining. At the later stage of ALS, the type II fibers usually either atrophy or convert to the type I fibers. Consistent with the previous study, our data showed that HS treatment regulated the dark blue staining in the gastrocnemius muscles of SOD1^G93A^ mice.

In conclusion, HS prolonged the lifespan and extended the disease duration in SOD1^G93A^ mouse model of ALS. HS also decreased the motor neuron loss and ameliorated the gastrocnemius muscles atrophy. The therapeutic effects of HS against ALS might be mediated by its activities of anti‐oxidative stress and anti‐inflammation through promoting transition of microglia polarization from M1 to M2 phenotype. All these findings predicted a promising therapeutic potential of HS for ALS.

## CONFLICT OF INTEREST

The authors declare no conflict of interest.
