# Supplementary figures and images for: Therapeutic effects of hirsutella sinensis on the disease onset and progression of amyotrophic lateral sclerosis in SOD1G93A transgenic mouse model
Source: CNS Neurosci Ther. 2019 Jul 18;26(1):90–100. doi: 10.1111/cns.13182 (PMC6930832; doi:10.1111/cns.13182)

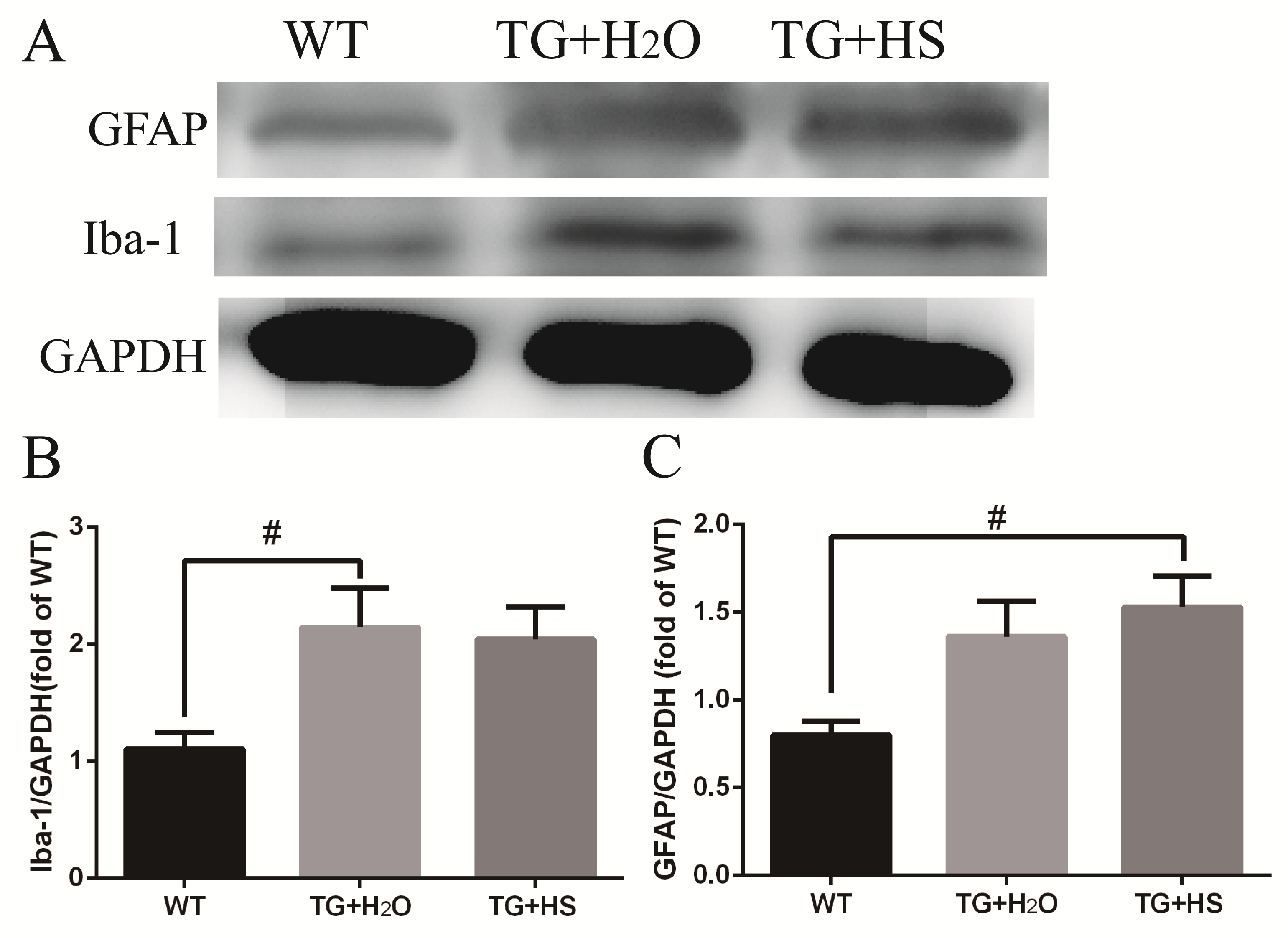

Supplement: Supplementary file 1 [file CNS-26-90-s001.png]
